# Evaluation of LncRNAs CBR3-AS1 and PCA3 expression in Gastric cancer and their correlation to clinicopathological variables

**DOI:** 10.18632/genesandcancer.241

**Published:** 2025-05-09

**Authors:** Parisa Najari, Sama Akbarzadeh, Ali Rajabi, Samaneh Tayefeh-Gholami, Elaheh Malek Abbaslou, Tooraj Ghasemzadeh, Mohammadali Hosseinpourfeizi, Reza Safaralizadeh

**Affiliations:** ^1^Department of Animal Biology, Faculty of Natural Sciences, University of Tabriz, Tabriz, Iran; ^2^Department of Biophysics, Istanbul Faculty of Medicine, Istanbul University, Istanbul, Turkey; ^3^Institute of Graduate Studies in Health Sciences, Istanbul University, Istanbul, Turkey

**Keywords:** GC, LncRNAs, *CBR3-AS1*, *PCA3*, qRT-PCR

## Abstract

Background: Gastric cancer (GC) is a multifactorial disease with a high death rate due to the unknown mechanisms involved in the developing, progressing, and late diagnosing GC. Several cancers have been linked to Long non-coding RNAs (lncRNAs), including GC, through differential expression. They play a crucial role in tumorigenesis pathways as modulatory factors, making them intriguing clinical and diagnostic biomarkers for many malignancies. This study’s objective is to compare the lncRNAs *CBR3-AS1* and *PCA3* expression levels in tumoral tissues to marginal tissues and the clinicopathological features of patients.

Methods and Results: 100 GC patients’ tumoral and marginal tissue samples from Tabriz’s Valiasr Hospital were gathered for this case-control research. To determine the expression level of *PCA3* and *CBR3-AS1* lncRNAs in GC, total RNA was extracted, and the qRT-PCR technique was employed. Compared to adjacent marginal tissues, the tumor tissue of patients with GC showed a significant increase in the expression levels of *PCA3* and *CBR3-AS1* (*P* < 0.0001). The expression ratio of lncRNA *CBR3-AS1* and *PCA3* did not significantly correlate with clinicopathological variables. The ROC curve’s findings lead to the conclusion that the genes lncRNAs *PCA3* and *CBR3-AS1*, with AUC values of 0.68 and 0.79, respectively, suggest that they could play carcinogenic roles in GC and may act as moderate diagnostic biomarkers for GC.

Conclusions: In GC, *CBR3-AS1* and *PCA3* may be utilized as therapeutic targets and prognostic biomarkers, respectively.

## INTRODUCTION

Gastric cancer (GC), a multifactorial disease, is affected by environmental and genetic factors and is frequently identified at advanced stages due to poor prognosis [1]. The fifth most typical malignant tumor worldwide was GC. 1.1 million additional cases and 800,000 fatalities are projected by GLOBOCAN 2020 [2]. Regular endoscopic screening, lowering H. pylori infection rates, targeted therapies, and immune therapies can affect the patient’s survival rate with GC; however, surgery is still the primary approach to treatment [3–5]. Numerous individuals deal with GC metastases because of the poor prognosis of the disease, making it crucial to identify biomarkers that contribute exclusively to the GC prognosis and target therapies [6]. Long non-coding RNAs (lncRNAs) are defined as non-coding RNAs comprising 200 nucleotides or longer [7]. These transcripts do not produce proteins and can regulate biological processes such as translation, posttranslational modifications, transcription, epigenetic alterations, cell division, proliferation, and stem cell pluripotency [8]. LncRNAs express abnormally in various tumors, some cancer-specific, and are found in plasma, urine, and body fluids, indicating disease severity [9]. Because lncRNAs may regulate GC cell proliferation, cycle, apoptosis, invasion, and metastasis, they may be useful as diagnostic and cancer treatment targets. This is due to their genome-wide expression patterns [10–14].

*PCA3* (prostate cancer antigen 3), or *DD3*, is a lncRNA controlled by steroid receptors that is transcription of 9q21.22. In 95% of prostate cancer cases, it is overexpressed, and both benign and malignant prostate cancer patients have it detected in their urine [15]. Through the process of adenosine deaminase, this gene controls the amounts of *prune2* in the body. *Prune2* is downregulated by *PCA3* overexpression, whereas *prune2* is upregulated by *PCA3* silence. In contrast, silencing of *prune2* caused a rise in cell proliferation in prostate cancer cells [16]. Other studies also show that this lncRNA regulates *p53* signaling and modulates the production of *miR-1261*, *miR-132-3p*, *PRKD3*, *SREBP1,* and *LAP2α*, all of which have critical implications on the development of prostate cancer [17].

lncRNA CBR3 antisense RNA 1 (*CBR3-AS1*) is situated in carbonyl reductase 3’s antisense region (*CBR3*), which is found in chromosome 21 (21q22.12) [18]. Research has indicated that *CBR3-AS1* stimulates the growth, migration, and invasion of cancer cells. As a potential oncogene in several malignant tumors such as gestational choriocarcinoma, lung, breast, cervical, and colorectal cancer, *CBR3-AS1* plays an important role [19–23]. As a result, *CBR3-AS1* has been proposed as a possible biomarker for cancer diagnosis and prognosis [19].

Considering the role of lncRNAs in cancer, this study aims to evaluate *PCA3* and *CBR3-AS1* expression levels and diagnostic biomarker values in patients with GC from Iran.

## RESULTS

### CBR3-AS1 and PCA3 expression levels in GC samples

**Figure 1:**
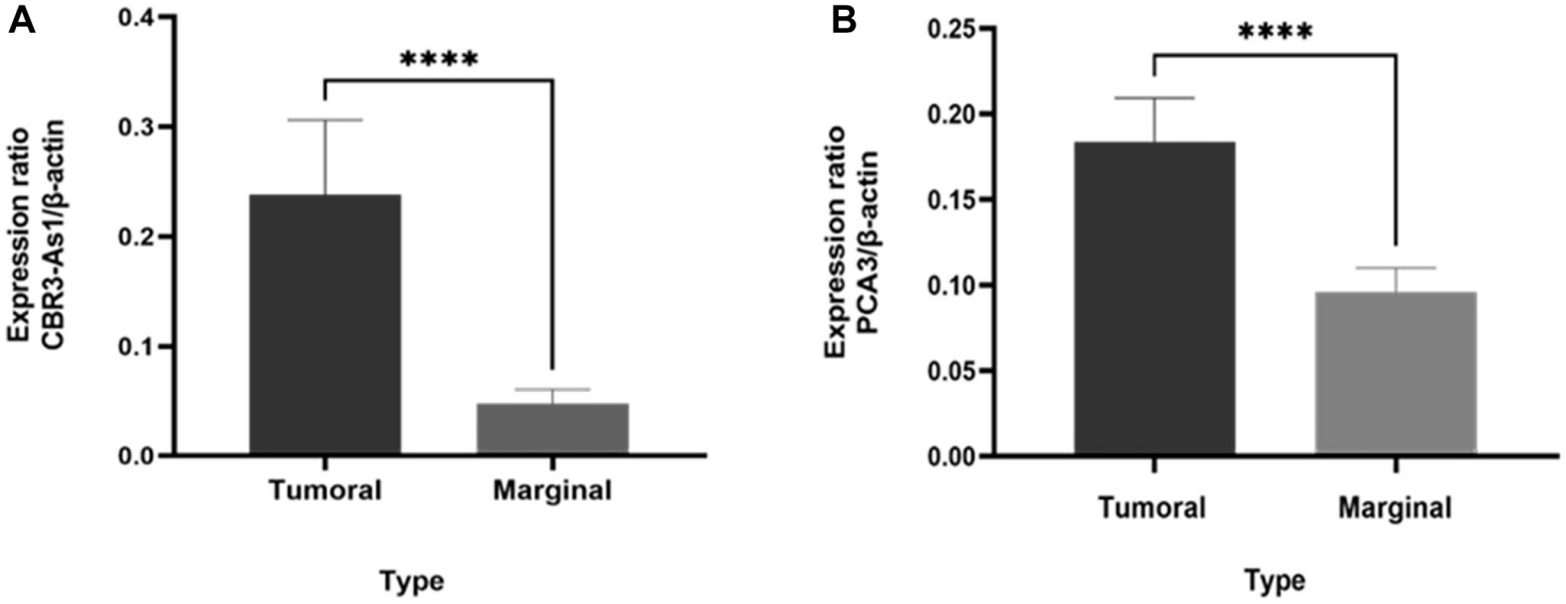
Comparison of *CBR3-AS1* and *PCA3* expression levels in patients with GC and marginal tissue. (**A**) *CBR3-AS1* (*P*-value < 0.0001), (**B**) *PCA3* (*P*-value < 0.0001).

The levels of *CBR3-AS1* and *PCA3* were evaluated in both cancerous tissues and the adjacent marginal tissues of 100 patients diagnosed with GC (Figure 1). Through statistical analyses, when comparing the expression levels in the tumor tissues to the adjacent marginal tissues, it was found that there was a noticeable overexpression (*p*-value < 0.0001).

### Association between lncRNA expression and clinical variables

In this study, the characteristics of GC patients were examined, specifically the correlation between the expression pattern of *CBR3-AS1* and *PCA3* and various clinicopathological features, such as age, tumor size, sex, TNM staging, H. pylori infection, lymph node metastasis, and histology. The findings indicate that no significant correlation was discovered between the expression levels of these lncRNAs and their respective traits of the clinicopathology. Table 1 provides a comprehensive overview of the relative expression ratios of the previously identified lncRNAs and subgroups.

**Table 1:** CBR3-AS1 and PCA3 expression and clinicopathological parameters of GC patients

Clinical parameters	*N*	Expression Mean LncRNA CBR3-AS1 Tumoral (±SD)	Expression Mean LncRNA CBR3-AS1 Marginal (±SD)	*P*-value	Expression Mean LncRNA PCA3 Tumoral (±SD)	Expression Mean LncRNA PCA3 Marginal (±SD)	*P*-value
Age				0.469			0.611
≤50	43	0.256 (0.358)	0.516 (0.067)		0.168 (0.201)	0.108 (0.157)	
>50	57	0.223 (0.325)	0.044 (0.060)		0.195 (0.286)	0.086 (0.126)	
Gender				0.471			0.87
Male	57	0.24 (0.384)	0.050 (0.067)		0.181 (0.224)	0.107 (0.157)	
Female	43	0.235 (0.271)	0.043 (0.057)		0.186 (0.287)	0.080 (0.114)	
Different tumor size				0.261			0.764
≤5 cm	30	0.155 (0.209)	0.057 (0.075)		0.195 (0.290)	0.091 (0.111)	
>5 cm	70	0.273 (0.377)	0.043 (0.057)		0.178 (0.236)	0.097 (0.152)	
Lymph involvement				0.88			0.558
Negative	27	0.261 (0.216)	0.043 (0.081)		0.199 (0.137)	0.094 (0.116)	
Positive	73	0.172 (0.373)	0.059 (0.055)		0.139 (0.283)	0.099 (0.149)	
TNM (Tumor stage)				0.87			0.69
Stage I and II	67	0.2044 (0.261)	0.0458 (0.060)		0.8308 (0.291)	0.083 (0.134)	
Stage III and IV	33	0.03054 (0.453)	0.0514 (0.068)		0.1220 (0.145)	0.122 (0.151)	
Helicobacter pylori infection				0.96			0.108
Positive	67	0.230 (0.304)	0.468 (0.067)		0.20 (0.261)	0.09 (0.138)	
Negative	33	0.251 (0.403)	0.493 (0.053)		0.148 (0.234)	0.106 (0.146)	
Lauren type				0.493			0.383
Intestinal	76	0256 (0.316)	0.046 (0.060)		0.170 (0.240)	0.101 (0.148)	
Diffuse	24	0.223 (0.398)	0.052 (0.073)		0.225 (0.286)	0.077 (0.112)	

### The ROC analysis

**Figure 2:**
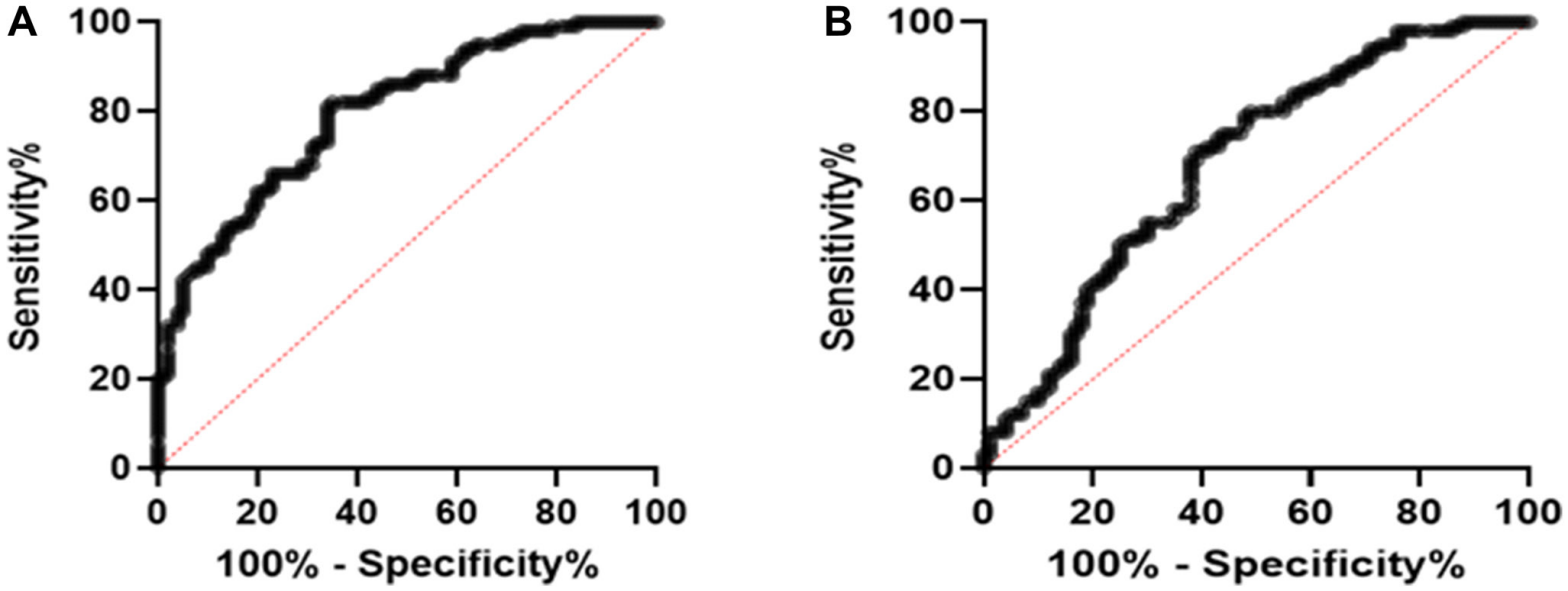
Roc covers the analysis of (**A**) *CBR3-AS1* (AUC = 0.79 (sensitivity = 81%, specificity = 66%, and 95% CI = 0.7359 to 0.8561)) and (**B**) *PCA3* (AUC = 0.68 (sensitivity = 69%, specificity = 62%, and 95% CI = 0.6086 to 0.7562)) to evaluate their biomarker potential in GC patients.

A ROC curve was constructed to assess the potential prognostic significance of *CBR3-AS1* and *PCA3* in individuals diagnosed with GC. The area under the curve (AUC) for *CBR3-AS1* was calculated to be 0.79 (with a sensitivity of 81%, specificity of 66%, and 95% CI = 0.7359 to 0.8561), while the AUC for *PCA3* was determined to be 0.68 (with a sensitivity of 69%, specificity at 62%, and 95% CI = 0.6086 to 0.7562). These findings suggest that *CBR3-AS1* and *PCA3* expressions are promising diagnostic biomarkers in GC patients (Figure 2).

### Correlation

**Figure 3:**
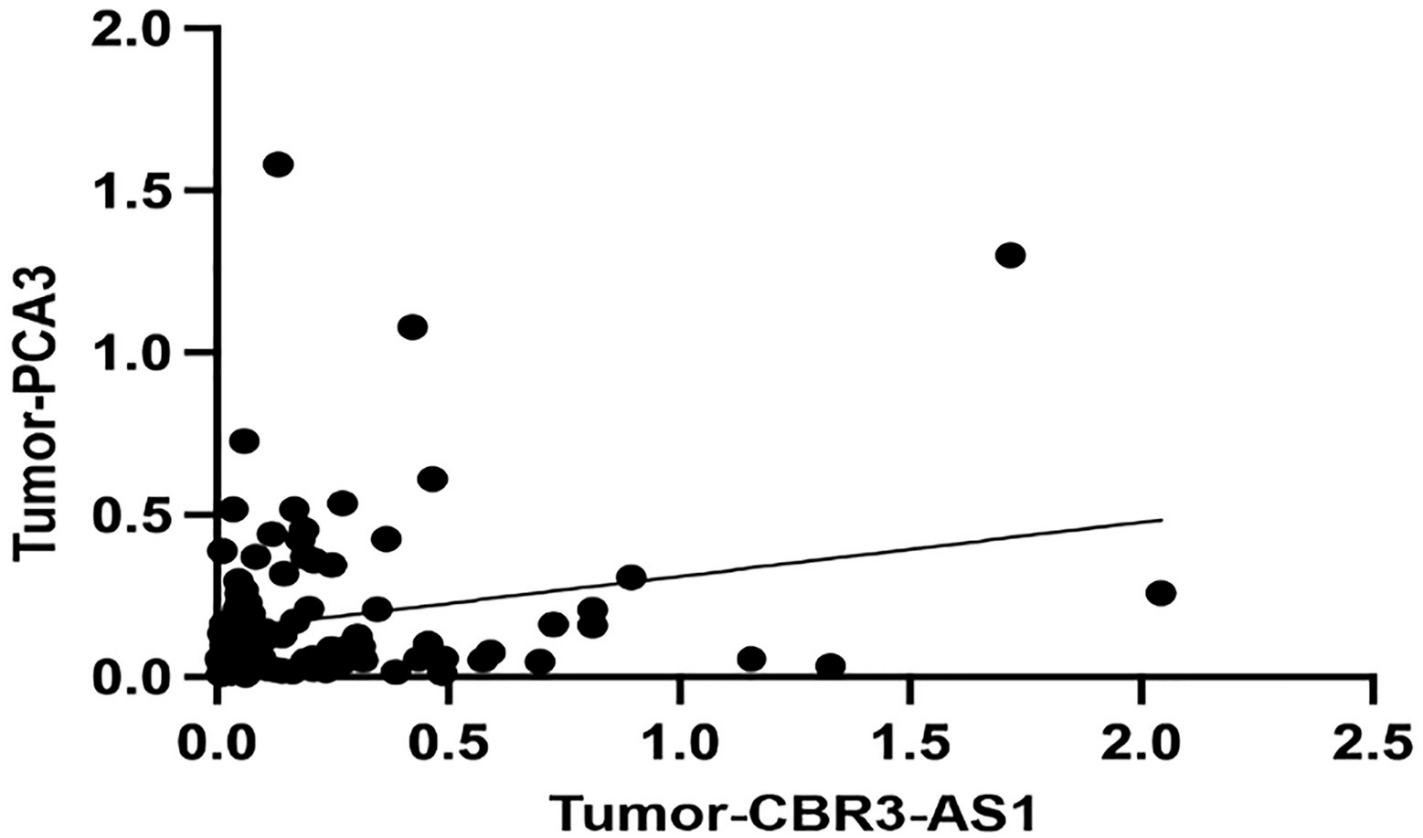
Correlations between the expression levels of *CBR3-AS1* and *PCA3* in GC patients (*r* = 0.1777, *P*-value = 0.0769).

There is no significant correlation between the expression of the *CBR3-AS1* and *PCA3* in GC patients (r = 0.1777, *P*-value = 0.0769), according to Spearman’s correlation analysis (Figure 3).

## DISCUSSION

GC is a multifactorial disease that poses a challenge to the oncology domain due to its high mortality rate and late diagnosis caused by a lack of effective biomarkers [24, 25]. Researchers show that the complex tumorigenesis may be connected to how lncRNAs interact with multiple biomolecules [26]. The current investigation demonstrates that *PCA3* and *CBR3-AS1* may play a carcinogenic role in individuals with GC since tumor tissue showed higher levels of *PCA3* and *CBR3-AS1* expression than marginal tissues. These findings suggest that both lncRNAs serve as moderate biomarkers for GC.

However, no significant correlation was found between the expression levels of CBR3-AS1 and PCA3 and clinicopathological parameters. This lack of correlation can be associated with several factors, including biological heterogeneity and small subgroup sizes within specific clinicopathological categories (Lymph involvement, TNM stages, and Lauren type). This may lead to variable gene expression patterns and prevent potential associations with clinical characteristics.

According to CAI Yi-Pin et al.’s research, cervical cancer tissue has a higher expression level of *CBR3-AS1*. They also suggest that the lncRNA *CBR3-AS1*/miR-3163/LASP1 pathway is critical in controlling the proliferation of cells and their stem cell-like characteristics [22]. According to another study that used qRT-PCR and western blot techniques, *CBR3-AS1* modifies the miR-140-5p/DDX54-NUCKS1-mTOR signaling pathway network, hence potentially contributing to an oncogenic function in osteosarcoma [27]. Xu L. and her colleagues detected that in tissues and cell lines from breast cancer, *CBR3-AS1* was upregulated and associated with a better prognosis [28]. Overexpression of *CBR3-AS1* in NSCLC tissues lowers proliferation, invasion, and migration while it increases apoptosis. *In vivo* tests revealed that *CBR3-AS1* accelerated tumor development and may control the activity of the miR-409-3p target gene SOD1. It has been noted that the *CBR3-AS1*/miR-409-3p/SOD1 pathway enables *CBR3-AS1* to play an oncogenic role [29]. Also, based on findings by Y Guan et al., upregulation of CBR3 AS1 has been linked to larger tumors, advanced TNM stages, lymph node metastases, and shorter survival periods. *CBR3-AS1* knockdown increased apoptosis and tumorigenicity while suppressing proliferation, migration, and invasiveness. It has been suggested that the *CBR3-AS1*/miR-509-3p/HDAC9 pathway is a valuable target for the therapy of NSCLC since it promotes tumor growth through the development and progression of NSCLC [30].

*PCA3* is a particular lncRNA for the detection of prostate cancer in a urine test approved by the FDA. By altering the expression of miR-132-3p, miR-1261, SREBP1, PRKD3, and LAP2, as well as controlling p53 signaling, this lncRNA has significant implications on prostate carcinogenesis [17, 31]. Liu Y et al. Studies discovered that the expression of the lncRNA *PCA3* was higher in epithelial ovarian cancer tissues than in healthy ovarian tissue. The siRNA-mediated knockdown of *PCA3* dramatically reduced cell proliferation, migration, and invasion. MiR-106b-5p may bind to the 3′UTR of *PCA3* and decrease the protein production of genes controlled by miR-106b; therefore, *PCA3* might be a useful diagnostic biomarker for treating epithelial ovarian cancer [32].

## MATERIALS AND METHODS

### Sampling of human gastric tissue

Endoscopy samples from 100 patients with stomach cancer were taken from their tumors and marginals at the Valiasr Hospital in Tabriz, Iran (total *n* = 200). Before surgery, no patient had undergone anti-tumor treatment as soon as the samples were resected. To extract the RNA, they were kept at −80°C and quickly frozen in liquid nitrogen. This investigation received clearance from the University of Tabriz’s Medical Ethics Committee and was conducted following the norms of Good Clinical Practice guidelines and the Declaration of Helsinki (approval number: IR. TABRIZU. REC. 1398.015). The histologic features of the samples were evaluated and categorized by a skilled pathologist. Every patient of GC signed an informed consent form.

### RNA extraction

Total RNA was extracted from the GC and adjacent marginal tissue samples using Trizol, as directed by the manufacturer. (Invitrogen, MA, USA). Thermo Fisher Scientific’s nanodrop spectrophotometer and 2% agarose gel electrophoresis were used to qualitatively and quantitatively evaluate the extracted RNAs. After that, every RNA sample was kept at −80 C until cDNA synthesis and DNaseI treatment were performed on them.

### cDNA synthesis and quantitative RT-PCR

A cDNA synthesis kit (Yektatajhiz, Iran) was used to synthesize complementary DNA (cDNA) from extracted RNA in accordance with the manufacturer’s instructions. Later, until the polymerase chain reaction (PCR), the synthesized cDNAs were kept at –20°C. The Gene Runner and Primer BLAST programs were used to design the oligonucleotide primers. To avoid producing non-specific PCR products, the primers were verified using the BLAST tool available on the NCBI website. The housekeeping gene β-actin was chosen as the baseline for normalizing gene expression. Table 2 displays the particular primers and product lengths for *CBR3-AS1*, *PCA3*, and β-actin.

**Table 2:** The primer sequences and length

Gene	Primer type	Sequences of primers
LncRNA CBR3-AS1	Forward	5′-GAGCGGGAGTCTTCCTTAGC-3′
	Reverse	5′-AGTTTTCCTTATGGGGTGTCCA-3′
LncRNA PCA3	Forward	5′-AGGGGAGATTTGTGTGGCTG-3′
	Reverse	5′-ATGTCCTTCCCTCACAAGCG-3′
β-actin	Forward	5′-AGAGCTACGAGCTGCCTGAC-3′
	Reverse	5′-AGCACTGTGTTGGCGTACAG-3′

The expression levels of *CBR3-AS1* and *PCA3* were measured by quantitative real-time reverse transcription-PCR (qRT-PCR), following the manufacturer’s instructions and utilizing Thermo Fisher Scientific’s Maxima SYBR-Green qRT-PCR master mix. The reaction mixtures, including 1 μL of cDNA (100 ng/μL), 5.4 μL of ddH2O, 0.6 μL of particular primers for *β-actin* (10 μM) and *CBR3-AS1*, and 14 μL of 7 μL of SYBR Green Master Mix (2×), were incubated at 95°C for 10 min. Subsequently, there were 40 amplification cycles carried out, with each cycle lasting 30 seconds at 95°C, 60°C, 72°C, and 72°C for 5 minutes. For *PCA3* and *β-actin*, the identical procedure was carried out again. Gene expression was measured using the 2−ΔCT computation, where CT was the threshold cycle.

### Statistical evaluation

The expression of lncRNAs was determined through qRT-PCR results utilizing the 2^−ΔCt^ method. The data were statistically analyzed using the Mann-Whitney *U*-test using GraphPad Prism 9. The Mann-Witney *U*-test and one-way ANOVA were used to investigate the association between the lncRNAs and clinicopathological features using SPSS (version 26). In addition, to analyze the correlation between two lncRNAs in tumoral tissues, the Spearman rho assay was performed using GraphPad Prism 9. The receiver operating characteristic (ROC) curve test was used to examine the biomarker potential of lncRNAs in GC patients using the GraphPad Prism 9 software. In every experiment, the *p*-value of less than 0.05 is deemed significant.

### Data availability statements

Data are available upon request.

## CONCLUSIONS

To sum up, this study focuses on the expression of CBR3-AS1 and PCA3 in GC tumoral and marginal healthy tissues. The expression levels of these genes were found to be remarkably higher in GC tumor samples compared to noncancerous marginal tissues. However, no significant correlation was found between the expression levels of both lncRNAs and the clinicopathological characteristics. Despite the promising diagnostic potential of CBR3-AS1 and PCA3 in GC, the current study has some limitations, including its single-center cohort, lack of functional validation assays (such as gene knockdown/overexpression, RNA-sequencing, and pathway analysis). Additionally, despite moderate AUC values, validation in independent patient cohorts is necessary to establish their reliability as biomarkers. Future studies should include multi-center validation, functional assays, and larger sample sizes to support our findings further and explore the underlying molecular mechanisms.
